# Ambulance service call handler and clinician identification of stroke in North East Ambulance Service

**DOI:** 10.29045/14784726.2021.09.6.2.59

**Published:** 2021-09-01

**Authors:** Graham McClelland, Emma Burrow

**Affiliations:** North East Ambulance Service NHS Foundation Trust ORCID iD: https://orcid.org/0000-0002-4502-5821; North East Ambulance Service NHS Foundation Trust

**Keywords:** emergency medical services, stroke, triage

## Abstract

**Introduction:**

Emergency medical services (EMS) are the first point of contact for most acute stroke patients. The EMS response is triggered by ambulance call handlers who triage calls and then an appropriate response is allocated. Early recognition of stroke is vital to minimise the call to hospital time as the availability and effectiveness of reperfusion therapies are time dependent. Minimising the pre-hospital phase by accurate call handler stroke identification, short EMS on-scene times and rapid access to specialist stroke care is vital. The aims of this study were to evaluate stroke identification by call handlers and clinicians in North East Ambulance Service (NEAS) and report on-scene times for suspected stroke patients.

**Methods:**

A retrospective service evaluation was conducted linking routinely collected data between 1 and 30 November 2019 from three sources: NEAS Emergency Operations Centre; NEAS clinicians; and hospital stroke diagnoses.

**Results:**

The datasets were linked resulting in 2214 individual cases. Call handler identification of acute stroke was 51.5% (95% CI 45.3–57.8) sensitive with a positive predictive value (PPV) of 12.8% (95% CI 11.4–14.4). Face-to-face clinician identification of stroke was 76.1% (95% CI 70.4–81.1) sensitive with a PPV of 27.4% (95% CI 25.3–29.7). The median on-scene time was 33 (IQR 25–43) minutes, with call handler and clinician identification of stroke resulting in shorter times.

**Conclusion:**

This service evaluation using ambulance data linked with national audit data showed that the sensitivity of NEAS call handler and clinician identification of stroke are similar to figures published on other systems but the PPV of call handler and clinician identification stroke could be improved. However, sensitivity is paramount while timely identification of suspected stroke patients and rapid transport to definitive care are the primary functions of EMS. Call handler identification of stroke appears to affect the time that clinicians spend at scene with suspected stroke patients.

## Introduction

Around 100,000 people have a stroke in the UK each year (Stroke Association, 2018) and the emergency medical services (EMS) are the first point of contact, and primary route into healthcare, for most acute stroke patients (Price et al., 2013). EMS response to stroke is triggered by patients, bystanders or other healthcare professionals contacting EMS, which in the UK is primarily via 999 or 111, where the call is triaged by call handlers and then an appropriate response, based on the nature of the call, is allocated by emergency medical dispatchers.

Early recognition of stroke symptoms is vital in triggering a rapid EMS response to minimise the time between onset of symptoms and definitive care (Rudd et al., 2020). The availability and effectiveness of reperfusion therapy, primarily thrombolysis but also thrombectomy, are time dependent, therefore EMS need to treat stroke as a time-critical condition due to the narrow window for acute treatment (Fassbender et al., 2013). When each minute saved equates to between 1.8 and 4.2 days extra healthy life (Meretoja et al., 2014; Meretoja et al., 2017), minimising the pre-hospital phase by accurate stroke identification by call handlers, short EMS on-scene times and rapid access to specialist stroke care is vital.

Previous research in this area has highlighted the range of performances of call handlers at identifying stroke, with a systematic review by Oostema et al. (2016) reporting 41–83% sensitivity and positive predictive values (PPV) of 42–68%. A large study reported similar sensitivity (66%) but a lower PPV (30%) using data from EMS Copenhagen, and concluded that failure to recognise stroke by call handlers could lead to delayed access to treatment and a negative effect on patient outcomes (Viereck et al., 2016).

Call handler recognition of stroke is important in triggering a timely response but it also affects the recognition of stroke and behaviour of EMS clinicians when they assess the patient face to face. Call handler recognition of stroke was shown to improve both the sensitivity (+15%) and PPV (+10%) of EMS clinician recognition of stroke (Mould-Milman et al., 2018). Call handler recognition of stroke has also been associated with shorter on-scene times (Odom et al., 2019; Oostema et al., 2018) and quicker transport to hospital (Caceres et al., 2013).

In this article, ‘call handler’ refers to staff based in the Emergency Operations Centre (EOC) assessing patients over the phone, whereas ‘clinician’ refers to staff assessing patients face to face, although the authors do recognise that clinical staff also work in the EOC.

### Aims

The aims of this study were to evaluate stroke identification by call handlers and clinicians in North East Ambulance Service NHS Foundation Trust (NEAS) and report on-scene times for suspected stroke patients.

## Methods

A retrospective service evaluation was conducted linking routinely collected data from three sources: NEAS EOC; NEAS clinicians; and hospital stroke diagnoses.

### Setting

The NEAS is the regional ambulance provider for around 2.7 million people in North East England covering Northumberland, Tyne and Wear, County Durham, Darlington and Teesside. NEAS employs around 2500 staff and manages over 1.5 million calls per year (NEAS, n.d.). Within the area covered by NEAS there are six hospitals receiving acute stroke patients.

NEAS clinicians primarily use the Face Arms Speech Test (FAST) (Harbison et al., 2003) for stroke identification and triggering admission to the nearest stroke unit. NEAS call handlers use NHS Pathways to support assessment of 999 and 111 calls and decision making. Call handlers identify stroke primarily based on FAST symptoms described by the caller. The NEAS EOC allocates disposition codes (Dx codes) to calls which are used to group calls together and reflect the category of response. Ambulances are dispatched using categories 1–4 which determines the priority of the response (NHS England, n.d.). Category 1 (C1) are immediately life threatening and receive the highest priority with a target response time of 7 minutes. Most stroke calls are category 2 (C2), which denotes a serious condition with an 18-minute response target. Category 3 (C3) calls are urgent issues with a 120-minute response target. Category 4 (C4) are non-urgent or stable problems which have a target response time of 180 minutes. Category 5 (C5) are dealt with remotely. Dx0117 is the code in the NHS Pathways system for ‘Emergency ambulance response for possible stroke time critical’ indicating stroke symptoms within four hours of onset, and this generates a Category 2 response. Dx0121 is a generic Category 3 code which would include call handler identification of stroke outside of the 4-hour onset window. Call handlers can also indicate stroke symptoms or consideration of stroke symptoms and arrive at a different disposition depending on the individual patient presentation.

### Data collection, extraction and analysis

Data were collected from three sources:

1. NEAS dispatch data from the NEAS EOC
2. NEAS clinician (primarily paramedic) data from the Electronic Patient Care Record (EPCR)
3. Patients admitted to hospital who were transported by NEAS with a confirmed stroke diagnosis identified from the Sentinel Stroke National Audit Programme (SSNAP).

Data were extracted by querying EOC and EPCR records for patients with any documentation of stroke. SSNAP data were supplied by the NEAS audit team. Data were manually linked using the NEAS call number which was available for all EOC and EPCR cases and the majority (99%) of SSNAP cases. The SSNAP cases without matching NEAS call numbers were probabilistically matched based on date and time of arrival, age, sex and home postcode. The three data sources were linked as described below:

1. Calls identified by call handlers as suspected stroke were linked with corresponding EPCR data
2. Cases identified by clinicians as suspected stroke on the ECPR were linked with corresponding call handler calls
3. Patients in SSNAP were linked with EPCR and call handler data.

Data extracted from the three sources included: sex; age; time of call; EOC disposition code; any call handler documentation of stroke; call to arrival time; arrival to leave time; leave to hospital time; call to hospital; destination; EPCR impression; inclusion in SSNAP; stroke type.

Data are described using descriptive statistics and sensitivity and PPV were calculated as measures of diagnostic accuracy. For calculating diagnostic accuracy values, patients included in SSNAP were used as the reference standard for true stroke identification.

### Timeframe

Data were collected from 1 to 30 November 2019. November was chosen to avoid the Christmas and New Year period and was pre-COVID.

## Results

In total 2261 unique cases were identified across all three datasets. Interhospital transfers (n = 46) and one case from the SSNAP report which was transported by Yorkshire Ambulance Service were removed. The final evaluation included 1910 cases identified from the call handler data, 718 cases identified from the EPCR data and 259 cases from the monthly SSNAP report (patients could be in all three datasets). The datasets were linked, resulting in 2214 individual cases from November 2019 who were identified as suspected stroke by NEAS call handlers or NEAS clinicians or who were included in the SSNAP report included in the evaluation.

**Figure 1:**
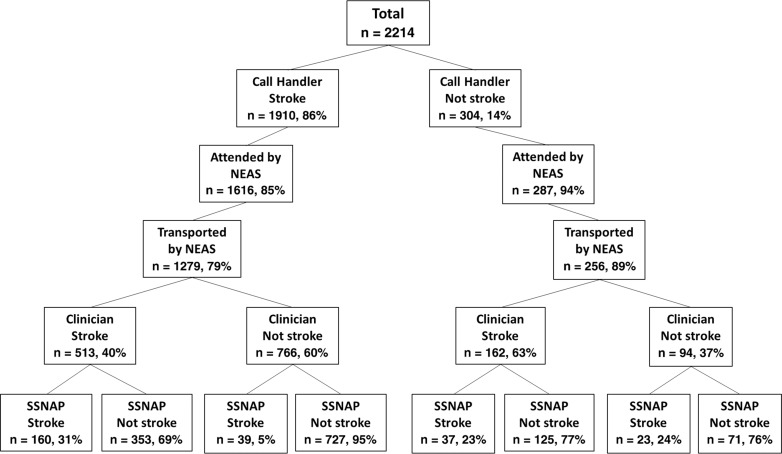
Figure 1. Potential stroke cases stratified by triage and SSNAP outcome.

Out of the 2214 total cases, 1903 (86%) patients were attended by NEAS, of whom 1535 (81%) were transported by NEAS to hospital (Figure 1).

Patients not attended by NEAS (n = 311, mean age 56.5 years (SD 22.3), 53% female) consisted primarily of non-emergency (C4 and C5, n = 234, 75%) dispositions and received advice or were referred over the phone. The majority of the emergency disposition calls (C2 and C3, n = 77, 25%) not attended by NEAS travelled to hospital via alternative means.

Patients attended by NEAS but not transported (n = 368, mean age 65.2 years (SD 20.6), 57% female) were mainly emergency (C1 and C2, n = 312, 85%) dispatches. The most common reasons for non-transportation documented on the EPCR were: patient refused hospital or interventions (n = 109, 30%); patient remained at home (n = 84, 23%); and symptoms improved or resolved (n = 70, 19%).

### Call handler identification of stroke

There were 1047 calls (55% female, mean age 70.5 years, SD 18.2) classified as Dx0117, and 134/1047 Dx0117 calls resulted in a patient documented as having a stroke in SSNAP. Therefore, call handler identification (based on Dx0117) of acute stroke was 51.5% (95% CI 45.3–57.8) sensitive with a PPV of 12.8% (95% CI 11.4–14.4)

Data were also examined where there was any recording of suspected stroke in the call handler record. There were 1910 calls (54% female, mean age 67.6 years, SD 19.4) where stroke was included in the call handler record, and 199/1910 calls resulted in a patient documented as having a stroke in SSNAP. Any call handler documentation of stroke was 76.8% (95% CI 71.2–81.8) sensitive with a PPV of 10.4% (95% CI 9.7–11.2).

The top 10 call handler disposition codes included in this evaluation that were not Dx0117 are shown in Table 1. The breakdown of call categorisation for all 2214 included cases is shown in Table 2. The majority (99%) of calls categorised as C4 and C5 were not attended by NEAS and were dealt with remotely.

**Table 1.** Non-Dx0117 call handler disposition codes.

Dx code	Explanation	N (%)
Dx0121	Emergency ambulance response (Category 3)	215 (18%)
Dx0112	Emergency ambulance response for acute coronary syndrome	211 (18%)
Dx0118	Emergency ambulance response for potential shock (adult/children)	129 (11%)
Dx11	Speak to primary care service within 1 hour (also override option in 111)	86 (7%)
Dx01124	Emergency ambulance response for level 2 health care professional request	78 (7%)
Dx01122	Emergency ambulance response for unconsciousness	73 (6%)
Dx108	Call is closed with no further action required	70 (6%)
Dx011	Emergency ambulance response	48 (4%)
Dx0101	Emergency ambulance response for potential cardiac arrest (purple R1)	47 (4%)
Dx05	To contact a primary care service within 2 hours	46 (4%)
	Others	164 (14%)

**Table 2.** Call categorisation by NEAS EOC.

Category	Call handler record included stroke	Dx0117 emergency response for stroke	Patients in SSNAP	Total
C1	12 (1%)	0	22 (8%)	87 (4%)
C2	1490 (78%)	1047 (100%)	215 (83%)	1671 (75%)
C3	189 (10%)	0	21 (8%)	220 (10%)
C4 & C5	219 (11%)	0	1 (<1%)	236 (11%)
Total	1910	1047	259	2214

### Clinician identification of stroke

There were 718 EPCRs (55% female, mean age 72.9 years, SD 15.1) where stroke was included in the impression, of which 675 patients were transported to hospital. Reasons for non-transport included: patient refusal (n = 14); symptoms improved/resolved (n = 11); referred to GP (n = 5); emergency healthcare plan in place (n = 4); remained at home (n = 3); making own way (n = 2); and others (n = 4). Out of the 718 EPCRs with impression including stroke, 367 (51%) were dispatched as Dx0117 and 537 (75%) had some mention of stroke in the call handler record.

Patients were linked with SSNAP records for 197/718 (27.4%) EPCRs including stroke in the impression, therefore any EPCR documentation of stroke was 76.1% (95% CI 70.4–81.1) sensitive with a PPV of 27.4% (95% CI 25.3–29.7). Examining cases where ‘stroke/TIA’ was the only recorded impression, there were 539 EPCRs and 179 matches with SSNAP, which results in a sensitivity of 69.1% (95% CI 63.1–74.7) and a PPV of 33.2% (95% CI 30.4–36.2).

### SSNAP patients

Patients included in the SSNAP report had a mean age of 75.0 years (SD 13.1), 51% were male and 88% were ischaemic strokes.

Comparing patients included in SSNAP with call handler identification showed that ischaemic strokes were more likely to have stroke in the call handler record (78% ischaemic vs 66% haemorrhagic), whereas Dx0117 was more likely to be recorded for haemorrhagic strokes (62%) than ischaemic strokes (50%).

Comparing patients included in SSNAP with clinician identification showed that haemorrhagic strokes were more likely to have stroke documented as an impression in the EPCR (any stroke impression, 83% haemorrhagic vs 75% ischaemic; stroke/TIA impression only, 72% haemorrhagic vs 68% ischaemic).

### Combined call handler and clinician identification of stroke

The sensitivity and PPV for combinations of call handler identification of stroke using Dx0117 and clinician recording of stroke on the EPCR are reported in Table 3.

**Table 3.** Diagnostic accuracy of combinations of call handler and EPCR identification of acute stroke.

Call handler Dx0117	EPCR impression includes stroke	Number of patients	Sensitivity (95% CI)	PPV (9% CI)
Positive	Positive	367	40.8% (34.7–47.0)	28.9% (25.1–32.9)
Positive	Negative	680	10.8% (7.3–15.2)	4.1% (2.9–5.8)
Negative	Positive	351	35.1% (29.3–41.3)	25.9% (22.2–30.1)

### On-scene times

The median on-scene times (n = 1535) for patients transported by NEAS were examined to see how dispatch category, call handler and EPCR identification of stroke affected the time spent with the patient prior to transport. On-scene times are reported in Table 4.

**Table 4.** On-scene times for patients transported by NEAS.

Patients	N	Median on-scene time (IQR)
All transported	1535	33 (25–43)
Dx0117	838	32 (24–42)
No Dx0117	697	35 (26–45)
EPCR impression includes stroke	675	32 (24–41)
EPCR impression does not include stroke	860	34 (26–46)
Dx0117 and EPCR both include stroke	348	30 (22–38)
No Dx0117 but EPCR includes stroke	327	35 (26–43)
C1 dispatch	82	34 (27–41)
C2 dispatch	1337	33 (25–43)
C3 dispatch	114	36 (30–48)

Call handler identification of acute stroke (Dx0117) appears to lead to shorter on-scene times, as does clinician recognition of stroke (EPCR documentation). The shortest on-scene times are reported when both call handlers and clinicians recognise stroke.

## Discussion

### Description of results

This study evaluated stroke identification in a UK ambulance service using routinely collected data and showed that call handlers identified between 52% and 77% of stroke patients but with a low (10–13%) PPV and that the sensitivity of clinician identification was 76% and they had a higher PPV of 27%. These results make sense, as the call handlers’ priority is to identify patients with potential stroke symptoms and rapidly dispatch an appropriate response (C2 or higher) so sensitivity is the most desirable characteristic, whereas clinicians assess the patient face to face so a higher PPV is to be expected.

When call handlers and clinicians both identified the patient as stroke the diagnostic accuracy was the highest (41% sensitivity, 29% PPV) and the on-scene times the shortest (30 minutes). Clinician identification of stroke appears to be more important than call handler identification of stroke in terms of combined accuracy. High priority dispatch (C1 and C2) resulted in a slightly shorter on-scene time than C3 dispatch.

### Results in context

The sensitivity of NEAS call handlers is in line with published figures but the PPV is lower than would be expected (Oostema et al., 2016; Viereck et al., 2016). The performance of NEAS clinician stroke identification in this evaluation was more sensitive but with a lower PPV than has been previously reported (McClelland et al., 2020). These differences could be due to the secondary use of a SSNAP report as the only reference standard in this evaluation, whereas previous work by the lead author has used more rigorous methods to establish final diagnoses.

The on-scene times reported here are higher, but in line with the upwards trajectory of previously reported figures for the same area (Haworth and McClelland, 2019). Patients identified by call handlers or NEAS clinicians as stroke had shorter on-scene times than those identified as other conditions. Call handlers and face-to-face clinicians both identifying the patient as stroke produced the shortest on-scene times (30 minutes), although these are still long compared to international standards (Drenck et al., 2019; Mosley et al., 2007; Schwartz et al., 2016).

A similar piece of work which focused on call handler identification of stroke conducted in East of England Ambulance Service using a different triage system (MPDS) found that 90% of 858 cases received a category 2 response, 51% of cases identified as stroke by clinicians were not identified as stroke by MPDS and that there were no significant differences in on-scene time or total job cycle time between cases identified as stroke and those identified as something else (Phillips et al., 2020). This NEAS evaluation reports similar categorisation, with 91% of true stroke cases receiving a C1 or C2 response, a similar proportion of disagreement between call handler and clinician stroke identification but a difference in on-scene time that was not present in the East of England data.

## Limitations and strengths

This was a retrospective service evaluation based on routinely collected data, therefore the results are not generalisable. The use of SSNAP as the only reference standard may lead to underestimation of the sensitivity and PPV of NEAS call handlers and clinicians, as SSNAP may not include 100% of stroke cases depending on when the data were extracted from SSNAP and exported to NEAS and the completeness of the SSNAP record. In this case, the number of patients in the SSNAP record (n = 259) is slightly lower than would be expected based on regional data but is in line with other months of SSNAP data sent to NEAS. The fact that stroke may be documented by call handlers but the final disposition and categorisation may not be Dx0117 reflects the uncertainties of telephone triage. It would require a large and focused piece of research to study the various ways that symptoms are reported by the person making the call, how these are then interpreted by ambulance service call handling systems and the resulting impact on EMS treatment of the patient such as the ESCORTT study (Jones et al., 2013).

## Implications for practice

Call handler and clinician identification of stroke in NEAS are both in line with other systems but there is room for improvement in both areas. On-scene times reported in this evaluation appear extended compared to other systems, and efforts need to be made to reduce these as even a 5-minute saving in the average call to hospital time would translate into improved patient outcomes, as described by Meretoja et al. (2014, 2017). While there are no treatments for stroke deliverable in a normal ambulance, increasing call handler and clinician diagnostic accuracy and reducing the call to hospital time are actions that will improve patient outcomes. Improved training and education, increased clinician awareness of on-scene time and its direct link to stroke patient outcomes and feedback on stroke cases may be ways of improving pre-hospital stroke care.

The call handler categorisation of suspected stroke calls affects the timeliness of the response to these patients, and with the time window for reperfusion treatment being extended by new studies and the increasing availability of mechanical thrombectomy, the roughly 10% of patients who do not receive a C1 or C2 dispatch may need to be looked at in order to ensure they are not being disadvantaged by waiting an extended time for an EMS response.

If specialist stroke responses such as mobile stroke units (Fassbender et al., 2017) are to be effectively utilised then careful thought needs to be given to which suspected stroke cases these units respond to and how call handlers allocate these limited resources.

## Implications for research

Improving the accuracy of call handler identification of stroke is an area where new technologies such as artificial intelligence or machine learning may offer opportunities. The impact of dispatch as stroke or as a non-stroke condition on clinician face-to-face assessment and behaviour is an area which could be explored to seek ways to improve patient care and reduce the time to definitive care in conditions such as stroke. This evaluation illustrates the type of information that can be generated by linking together routinely collected datasets which could be done on a larger, potentially national scale, to inform future research and service improvement.

## Conclusion

This service evaluation using ambulance data linked with national stroke audit data showed that the sensitivity of NEAS call handler and clinician identification of stroke is similar to figures published on other systems but the PPV of call handler and clinician identification could be improved. However, sensitivity is more important in the current system where timely identification of suspected stroke patients and rapid transport to definitive care are the primary functions of EMS. Call handler identification of stroke appears to affect the time that clinicians spend at scene with suspected stroke patients.

## Acknowledgements

Thanks to the NEAS audit team for their support. Thanks to Chris Ashton and the pre-hospital stroke task and finish group for their support. Thanks to Professor Chris Price for his advice and support.

## Author contributions

GM conceived and designed the study and analysed the data. EB extracted and linked the data. Both authors read and approved the final manuscript. GM acts as the guarantor for this article.

## Conflict of interest

GM is on the editorial board of the BPJ.

## Ethics

This study was conducted as a service evaluation, therefore Health Research Authority and ethical approvals were unnecessary. The study was registered and approved by NEAS Research and Development department on 26 May 2020, ref. NEAS-SE-01-2020.

## Funding

GM is funded by the Stroke Association on a post-doctoral fellowship.
